# Supplementary material for: Syntactic- and morphology-based text augmentation framework for Arabic sentiment analysis
Source: PeerJ Comput Sci. 2021 Apr 5;7:e469. doi: 10.7717/peerj-cs.469 (PMC8049132; doi:10.7717/peerj-cs.469)
Supplement: Supplemental Information 4 [file peerj-cs-07-469-s004.pdf]

Raw Data
This code is shared using FigShare with the following URL and DOI

https://figshare.com/s/2fd1b7f1a6c91f659a3c

DOI:
10.6084/m9.figshare.14074268
